# A standardized polyherbal preparation POL-6 diminishes alcohol withdrawal anxiety by regulating *Gabra1, Gabra2, Gabra3, Gabra4, Gabra5* gene expression of GABA_A_ receptor signaling pathway in rats

**DOI:** 10.1186/s12906-020-03181-2

**Published:** 2021-01-06

**Authors:** Lalit Sharma, Aditi Sharma, Ashutosh Kumar Dash, Gopal Singh Bisht, Girdhari Lal Gupta

**Affiliations:** 1 Department of Pharmacy, Jaypee University of Information Technology, Waknaghat, Solan, Himachal Pradesh 173234 India; 2 School of Pharmaceutical Sciences, Shoolini University, Solan, Himachal Pradesh 173229 India; 3 Natural Product Chemistry Division, CSIR-Indian Institute of Integrative Medicine, Jammu, 180001 India; 4 Department of BT/BI, Jaypee University of Information Technology, Waknaghat, Solan, Himachal Pradesh 173234 India; 5 Department of Pharmacology, Shobhaben Pratapbhai Patel School of Pharmacy & Technology Management, Shri Vile Parle Kelavani Mandal’s Narsee Monjee Institute of Management Studies University, Mumbai, Maharashtra 400056 India; 6 School of Pharmacy & Technology Management, Shri Vile Parle Kelavani Mandal’s Narsee Monjee Institute of Management Studies, Shirpur Campus, Shirpur, Maharashtra 425405 India

**Keywords:** Polyherbal preparation, Alcohol withdrawal, Anxiety, GABA_A_ receptor, Gene expression

## Abstract

**Background:**

Alcohol abuse is a major problem worldwide and it affects people’s health and economy. There is a relapse in alcohol intake due to alcohol withdrawal. Alcohol withdrawal anxiety-like behavior is a symptom that appears 6–24 h after the last alcohol ingestion.

**Methods:**

The present study was designed to explore the protective effect of a standardized polyherbal preparation POL-6 in ethanol withdrawal anxiety in Wistar rats. POL-6 was prepared by mixing the dried extracts of six plants *Bacopa monnieri, Hypericum perforatum, Centella asiatica, Withania somnifera, Camellia sinesis,* and *Ocimum sanctum* in the proportion 2:1:2:2:1:2 respectively. POL-6 was subjected to phytochemical profiling through LC-MS, HPLC, and HPTLC. The effect of POL-6 on alcohol withdrawal anxiety was tested using a two-bottle choice drinking paradigm model giving animals’ free choice between alcohol and water for 15 days. Alcohol was withdrawn on the 16th day and POL-6 (20, 50, and 100 mg/kg, oral), diazepam (2 mg/kg) treatment was given on the withdrawal days. Behavioral parameters were tested using EPM and LDT. On the 18th day blood was collected from the retro-orbital sinus of the rats and alcohol markers ALT, AST, ALP, and GGT were studied. At end of the study, animals were sacrificed and the brain was isolated for exploring the influences of POL-6 on the mRNA expression of GABA_A_ receptor subunits in the amygdala and hippocampus.

**Results:**

Phytochemical profiling showed that POL-6 contains major phytoconstituents like withaferin A, quercetin, catechin, rutin, caeffic acid, and β-sitosterol. In-vivo studies showed that POL-6 possesses an antianxiety effect in alcohol withdrawal. Gene expression studies on the isolated brain tissues showed that POL-6 normalizes the GABAergic transmission in the amygdala and hippocampus of the rats.

**Conclusion:**

The study concludes that POL-6 may have therapeutic potential for treating ethanol-type dependence.

## Background

Alcohol is amongst the most broadly utilized and abused drugs [1]. According to the World Health Organization (WHO) report, 76.3 million individuals over the world have alcohol use disorders and reliance which results in 1.8 million deaths each year. Alcohol withdrawal syndrome is a state that shows up after a decrease or sudden end of consistent heavy drinking in individuals experiencing alcohol dependence [2]. Abstinence from chronic alcohol consumption prompts over-excitation of glutamatergic neuronal synaptic transmission in the amygdala, which results in anxiety characterized by a raised negative emotional response. Negative enthusiastic reactions originating from ethanol withdrawal lead to the resumption of alcohol drinking which is associated with intense craving and desire to take ethanol. Ethanol withdrawal anxiety generally shows up within 6–24 h of restraint from alcohol [3]. Alcohol withdrawal manifestations may be marked by seizures, heart failure and death happen in 5 to 10% of patients [4]. Alcohol withdrawal anxiety works as an unconditioned stressor for invigorating unconditioned withdrawal reactions, which prompts the actuation of a few cerebrum areas, particularly the regions that are engaged with the tweak and articulation of anxiety-like behaviors, for example, the amygdala, hippocampus, prefrontal cortex, and hypothalamus. Among the different limbic structures, the amygdala and hypothalamus are believed to have an imperative role in modulating ethanol withdrawal anxiety-like behaviors [5]. Ethanol utilization prompts neurobiological and behavioral alterations which are intervened by GABA_A_ (γ-aminobutyric acid) inhibitory receptor frameworks. Ethanol reliance results in a diminished GABA_A_ neuroreceptor response, this regulates ethanol drinking reinforcement reward, resilience, dependence, and withdrawal. GABAergic frameworks can be the imperative medication that focuses on accomplishing long haul forbearance from alcohol and alcohol withdrawal anxiety [6]. At present treatment choices for alcohol withdrawal anxiety are not many, for example, benzodiazepines are the main medication, yet their use is associated with sedation, psychological hindrance and addiction. Subsequently, the assurance of new powerful and therapeutically beneficial medications for the treatment of alcohol withdrawal anxiety is imperative [7].

Plants are the important sources of medication and a large number of drugs being used are derived from plants [8]. Polyherbal preparations have accomplished wide recognition in contrast with unrefined plant extracts and are broadly utilized for treating different disorders because of convenience, decrease in dose proportion, and simplicity of administration. A large portion of the synthetic medications gives symptomatic alleviation by following up on a solitary molecular target although the multi-target action of polyherbal preparations is helpful in interminable conditions. It ought to be noticed that herbal preparations have been esteemed for their additional viability because of the synergistic impact of numerous herbs [9]. *Hypericum perforatum* Linn (St. John’s wort), family Hypericaceae is reported to have anti-anxiety, antidepressant, pain-relieving, and other mood disorders related properties [10]. *Bacopa monnieri* (L.) Pennell is usually known as Brahmi family Scrophulariaceae is accounted for to have defensive impacts against neurological disorders like epilepsy, depression, stress, insanity, psychosis, and sleep deprivation [11]. *Centella asiatica* (L.) Urban (Gotu kola) family Apiaceae is accounted for to have different pharmacological activities like an antiepileptic, antidepressant, nervine tonic, rejuvenant, sedative, and tranquilizer [12]. *Withania somnifera* (L.) Dunal is usually known as Ashwagandha family Solanaceae is professed to have antianxiety, pain-relieving, antistress, and anti-inflammatory properties [13]. *Ocimum sanctum* Linn. commonly known as Tulsi family Lamiaceae has pain-relieving, antistress, antipyretic, anticonvulsant, neuroprotective, immunomodulatory, calming, and memory enhancer properties [14]. *Camellia sinensis* (L.) Kuntze is commonly known as Green tea family Theaceae have pharmacological properties like anti-Parkinson, anti-aging, antistroke, and anti-Alzheimer’s [15]. These plants have diverse pharmacological properties and have been utilized for the preparation of polyherbal preparations in the Ayurveda system of drugs for treating many ailments. In previous investigations, it has been discovered that a blend of *Camellia sinensis*, *Bacopa monnieri,* and *Hypericum perforatum* have synergistic antioxidant properties [16]. Owing to the fact of synergistic activities and diverse pharmacological effects of these medicinal plants, a polyherbal preparation (POL-6) comprising six plant extracts *Withania somnifera, Camellia sinesis*, *Hypericum perforatum, Centella asiatica, Bacopa monnieri,* and *Ocimum sanctum* was developed and standardized in our previous studies [17]. In other studies as per OECD 423 guidelines for safety evaluation, POL-6 showed that it is non-toxic. In acute toxicity studies 2000 mg/kg and in 28 days repeated oral toxicity studies 1000 mg/kg were considered as the no observed adverse effect levels (NOAEL) of POL-6 [18]. The present investigation was designed to evaluate the pharmacological influences of POL-6 on ethanol withdrawal anxiety-like behavior and its effects on the gene expression changes in the amygdala and hippocampus of the rats.

## Methods

### Plant material

The collection of *Hypericum perforatum* L. aerial parts was done from the herbal garden of the Jaypee University of Information Technology, Himachal Pradesh (H.P.), India and validated by Dr. Yashwant Singh Parmar University of Horticulture and Forestry, Solan, H. P. (Field book number: 13420). The dried leaves of *Camellia sinensis* (Batch No. ERM–23) and *Ocimum sanctum* (Batch No. RHD 283), dried roots of *Withania somnifera* (Batch No. EBD–18), and dried entire plant of *Centella asiatica* (Batch No. ERD–040) and *Bacopa monnieri* (Batch No. ERD–92) were procured from Natural Remedies, Bangalore, India. The fresh material from six plants was washed, shade dried cut into small pieces individually pulverized to form a coarse powder (confirmed by passing through sieve number 60). The coarse powdered individual plant material was placed in soxhlation assembly and defatted with pet. Ether (30–40 °C). After defatting, plant material was air-dried and then further exposed for 48 h at 50 °C using hydro-alcoholic extraction (70% v/v). The obtained solvent was then removed using a rotatory evaporator. The semisolid mass obtained after rotatory evaporation was further lyophilized to obtain dry powder and was refrigerated at 2–8 °C individually for further use. The dried powdered extract of the plants (*Bacopa monnieri, Hypericum perforatum, Centella asiatica, Withania somnifera, Camellia sinesis, and Ocimum sanctum*) was weighed independently and blended in the ratio (2:1:2:2:1:2) respectively utilizing a twofold cone blender. To get a homogeneous mix blend was further sieved to mesh size 40 and kept in a firmly clean container (closed) away from heat, moisture until further use [17].

### Liquid chromatography-mass spectroscopy profiling of POL-6

The instrument used for LC-MS was MicroTOF-Q and the technique used was ESI (Electrospray ionization technique). Phenomenex C18 (150 × 4 mm i.d., 5 μ) with a single quadrupole mass spectrometry analyzer was used for the liquid chromatography separation. An amount of 0.5% formic acid–acetonitrile (75:25%) was used as the mobile phase. The flow rate was 0.5 ml/min. The solvent was controlled by isocratic elution. The column temperature was kept at 30 °C. The MS spectrum was gained in the positive ion mode and was scanned from 50 m/z-1000 m/z. The Nebulizing pressure of the drying gas (N_2_) was 25 psi, the temperature was 350 °C at a gas flow rate of 6 ml/min. About 0.5 g of POL-6 was diluted with methanol and filtered with a 0.22 μm nylon filter before the examination. A 5 μl volume of the POL-6 was injected onto the column for examination. The mass fragmentations were identified by using a spectrum database for organic compounds [19].

### Qualitative phytochemical profiling of POL-6 by HPLC

POL-6 (5 mg) was dissolved in 10 mL methanol (80%) and 25 ppm solution was made by diluting it further. The solution was filtered with a 0.22 μm syringe filter. The HPLC system (Agilent Technologies) consists of an LC-binary pump, diode array detector, EZ-chrom system controller, and Innoval C18 (4.6 × 250 mm) column. For separation, 0.14 g of anhydrous potassium dihydrogen orthophosphate (KH_2_PO_4_) was added to 900 ml of HPLC grade water and orthophosphoric acid (0.5 ml) was dissolved above the mixture. The volume of the mixture was made upto1000 ml with water and then filtered through a membrane filter (0.45 μ). After filtration, the solution was placed in a sonicator for 3 min. The prepared solution was considered as the mobile phase gradient solvent (A) and the Acetonitrile was used as the solvent (B). An amount of 20 μl sample was injected through the SIL-HTC Shimadzu Autosampler. The conditions for the solvent system were 20:80 ratios, flow rate of 1.5 ml/min, and a run time of approximately 45 min. The chromatogram was obtained at a wavelength of 227 nm [20].

### HPTLC quantification of major constituents in POL-6

HPTLC instrument with CAMAG Linomat V automatic sample applicator, TLC scanner III, Camag twin trough chamber 10 × 10 cm, and WinCATS software was used for the HPTLC study. POL-6 (suspended in methanol) and standard solutions (each 5 μl) (suspended in methanol) were applied in the form of a band having bandwidth 8 mm; distance between the bands 14 mm and a constant application rate of 150 nL s^− 1^ using a microsyringe (Hamilton-Bonaduz Schweiz, Linomat syringe, 500 μl size) to a silica gel pre-coated 60 F254 TLC plates (10 × 10 cm with 200 μm thickness). TLC plates were then placed under the different mobile phases in a glass developing chamber and development was performed in an ascending manner to a distance of 8 cm. After the development, the densitometric scanning of the air-dried plate was performed with the help of a TLC scanner operated in reflectance-absorbance mode, slit dimensions: 6 × 0.45 at 254 nm. The calibration curve of all the standards was drawn. The sample and standard spots were applied on TLC plates and the contents of metabolites were analyzed using the regression equation from the calibration plot and expressed as % w/w [21].

### Animals and housing

Wistar rats of either sex were bought from the NIPER, Punjab, India, and housed at Animal House, Jaypee University, Solan, H.P. They were acclimatized to laboratory conditions kept at temperature 23 ± 2 °C, light and dark cycle (12:12 h). Animals were sustained with nutritional pellets diet (Aashirwad Industries, Chandigarh, India) and water ad libitum. The protocol was duly approved by IAEC, Jaypee University, Solan, H.P. India (3/GLG/2014/JUIT/IAEC). It was conducted in strict compliance with internationally accepted principles for laboratory animal use and care and as per the guidelines by the Committee for the Purpose of Control and Supervision of Experiments on Animals standards (1716/PO/a/13/CPCSEA) conforming ARRIVE guidelines for research on animals.

### Ethanol withdrawal study

#### Experimental design

Animals were divided randomly into 6 groups (*n* = 6) and housed separately. Group 1 received a liquid diet; Group 2 was subjected to voluntary ethanol intake for 15 days and received the vehicle 0.5% carboxymethyl cellulose (CMC) during ethanol withdrawal days (16th, 17th^,^ and 18th day). Groups 3, 4, and 5 were given alcohol treatment for 15 days and during ethanol, withdrawal days received POL-6 (20, 50, and 100 mg/kg, *p.o.*) once a day, respectively. Group 6 received alcohol treatment for 15 days and during the ethanol withdrawal period diazepam (2 mg/kg, *p.o.*) was administered once a day. Alcohol treatment was given to the animals as described in the earlier studies [22]. The alcohol-fed animals were allowed to have a free intake of 4.5%v/v ethanol on the 1st day, 7.5%v/v ethanol on the 2nd day, and 9% v/v ethanol from the third day to 15th day with a 2 bottle choice paradigm (water vs ethanol). On the 16th day, alcohol was withdrawn and a liquid diet (alcohol-free) was introduced to the alcohol-fed animals while control group animals were continued on the same diet. As per earlier studies, the peak level of anxiety was observed on the 3rd day of withdrawal i.e. on the 18th day. Hence animals in the present study were subjected to the behavior parameters analysis on day 18th only [23]. One hour after the last dose of the drug treatment animals were individually tested for examining the anxiety on the elevated plus-maze and light-dark test. After behavior studies blood was withdrawn immediately through retro-orbital of rats and serum was separated for the examination of biochemical parameters. Bodyweight change and ethanol intake of the animals were recorded every day during the study and expressed as g/kg/day. After completion of the study, animals were sacrificed by cervical dislocation and the brain was isolated. The amygdala and hippocampus were isolated from the rat brain for further analysis of variations in mRNA expression by RT-PCR [24, 25].

#### Blood alcohol concentration measurement

After consistent deliberate ethanol intake by the animals for 15 days blood was collected from the tail vein on day 15th and 16th, 17th^,^ and 18th day (alcohol withdrawal period) into EDTA coated vials. Blood containing vials were then centrifuged (1500×g) at 4 °C for 5–8 min. Plasma was stored at − 20 °C. BAC was determined by using bioassay systems’ EnzyChrom ethanol assay kit [26].

### Behavioral tests

#### Elevated plus maze test

EPM is a widely used test for studying anxiolytic responses in rats. Rats have an aversion for open and high space and prefer to live in the enclosed arm, when a rat is exposed to an open arm there is fear like movements and they freeze. The model is elevated at a height of 50 cm consists of a central platform with two open arms crossed with two closed arms. The rat was placed separately in the central compartment with the head facing towards the open arm. The parameters that were evaluated were (a) Time spent and number of entries in the open arms b) Time spent and the number of entries was counted in the closed arms with four paws) were recorded for 5 min [27].

#### Light and dark model test

The model comprises two plexiglass compartments one light (30 × 30 × 35 cm; 100 lx illumination) and one dark (20 × 30 × 35 cm; 40 lx illumination) connected by an opening of 7.5 × 7.5 cm in the middle of the divider. The rat was put separately in the middle of the light chamber having their back towards the dark compartment. The number of transitions between the light and dark compartment and the time spent in the light and dark chamber was recorded for 5 min [28].

#### Biochemical examination

For biochemical examination, blood was collected from the retro-orbital sinus of the rats on the 18th day. Blood was collected in Ethylene-diamine-tetraacetate (EDTA) coated tubes for mean corpuscular volume (MCV) determination. Blood was also allowed to clot for 30 min and then clotted blood tubes were centrifuged at 3000 RPM, 4 °C temperature for 10 min, and the acquired serum was utilized to access biochemical parameters like ALT, AST, ALP, and Gamma-glutamyltransferase (GGT) through commercially available kits [23].

### Real-time quantitative polymerase chain reaction

On completion of the study protocol rats were sacrificed by cervical dislocation method. The amygdala and hippocampus were extracted from the whole brain using an adult rat brain matrix (Kent Scientific, USA) and placed in a sterile tube containing RNAlater solution (5 volumes). The tubes containing the brain tissues were further stored at -80 °C until further analysis [29]. RT-PCR was carried out for studying mRNA expression of GABA_A_ receptor subunits namely Gabra1, Gabra2, Gabra3, Gabra4, and Gabra5. Trizol reagent was used for extracting total RNA from the brain tissues. RNA purity was evaluated on agarose gel (1.5%) in gel electrophoresis further quantification was done using a Nanodrop spectrophotometer (Thermo Scientific). Further 2 μg of total RNA was used for reverse transcription utilizing a verso RNA-to-cDNA synthesis kit (Thermo scientific). The RT-PCR analysis was performed using predesigned gene-specific primers for GABA_A_ receptor subunits using Primer Quest Tool (Gabra1 FP: GCCCTCCCAAGATGAACTTA, RP: AGTTACACGCTCTCCCAAGC; Gabra2 FP: ACCTTCTTTCACAACGGGAA, RP: GGAAAGTCCTCCAAGTGCAT; Gabra3 FP: ACCTTCTTTCACAACGGGAA, RP: CAGTCACTGCATCTCCAAGC; Gabra4 FP: CCGTATCCTGGACAGTTTGC, RP: ACATCAGAAACGGGTCCAAA; Gabra5 FP: TGAGACCAATGACAACATCA, RP: TAGATGTCTGTTCGCACCTG and GAPDH FP: TTCACCACCATGGAGAAGGC, RP: GGCATG GACTGT GGTCAT GA). Bio-Rad CFX96™ RT-PCR detection system was used for RT-PCR amplification using SYBR green dye. Total reaction mixer volume was 12.5 μl comprising 2.5 pM of each primer and cDNA template (1 μl). Relative expression levels of the target genes were estimated using housekeeping gene rat GAPDH as an endogenous control. The thermal cycle profile for 40 cycles to amplify cDNA was as 95 °C for 3 min; 95 °C for 15 s, 50 °C – 57 °C for 60 s, and 72 °C for 2 min. The gene expression of the targeted genes was calculated by using the double delta threshold cycle (ΔΔCT) method [30, 31].

### Statistical analysis

GraphPad Prism software 8.0 was used for statistically scrutinizing the data and was expressed as the mean ± SEM (standard error of mean). Two way ANOVA was used for analyzing the data followed by Dunnett’s multiple comparison post hoc test with a confidence level of *p* < 0.05.

## Results

### Liquid chromatography-mass spectroscopy profiling of POL-6

**Fig. 1:**
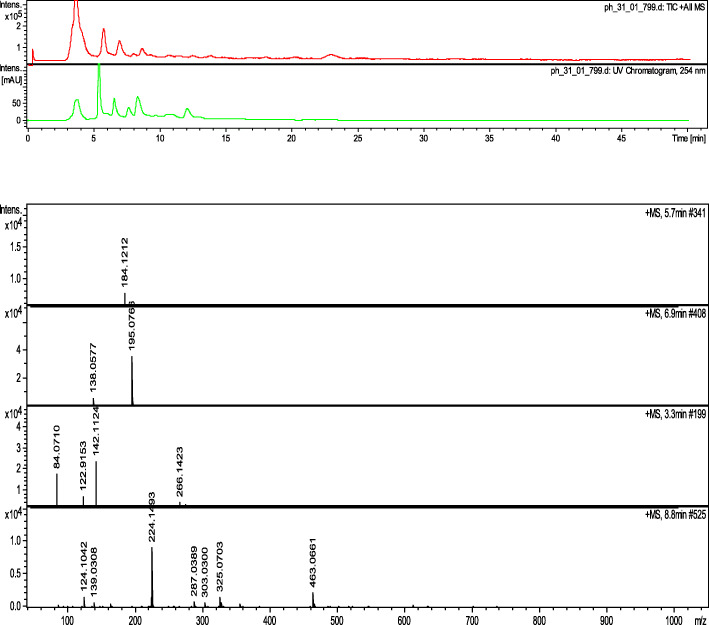
LC-MS spectrum of POL-6

We initiated our investigation with a mass analysis of the POL-6. The chemical constituents present in the POL-6 were recognized using liquid LC-MS spectroscopy (Fig. 1). We observed numerous peaks and each peak were analyzed further. Among all peaks, an intense peak was observed at 291.0898 m/z. When we matched masses of various natural products in the database (Dictionary of Natural Product 28.2), we found it, almost similar to catechin (Expected mass + H^+^: 291.0791). Hence the first confirmed compound present in the POL-6 was Catechin. Similarly, 12 more compounds were identified and summarized (Table 1). The identified compounds were Withaferin A, Withanolide, Luteolin, β-Setosterol, Quercetin, Hypericin, Rutin, Linalool, Caffeic acid, Catechin, Eugenol, D-Mannitol, and Withanone. But the discrepancy of this method was a similarity in masses of Withaferin A, Withanolide, and Withanone. For more authentications, we carried out the HPLC profiling of POL-6 further.

**Table 1:** Identified compounds and their expected and observed masses

Sr. No.	Compound As In Spectra	Expected Mass (g/mol)	Observed Mass (g/mol)
1.	Withaferin A+ H^+^	471.2740	471.2686
2.	Withanolide+ H^+^	471.2740	471.2686
3.	Luteolin+ H^+^	287.0346	287.0355
4.	β-Setosterol+ H^+^	415.6800	415.6806
5.	Quercetin + Na^+^	325.0236	325.0721
6.	Hypericin+ H^+^	505.0920	505.1420
7.	Rutin+ H^+^	611.1301	611.1354
8.	Linalool+ H^+^	155.1430	155.1432
9.	Caffeic acid+ H^+^	181.1200	181.1210
10.	Catechin+ H^+^	291.0860	291.0898
11.	Eugenol+ H^+^	165.0910	165.0918
12.	D-Mannitol+ Na^+^	205.0680	205.0402
13.	Withanone+ H^+^	471.2740	471.2686

### Qualitative phytochemical profiling of POL-6 by HPLC

**Fig. 2:**
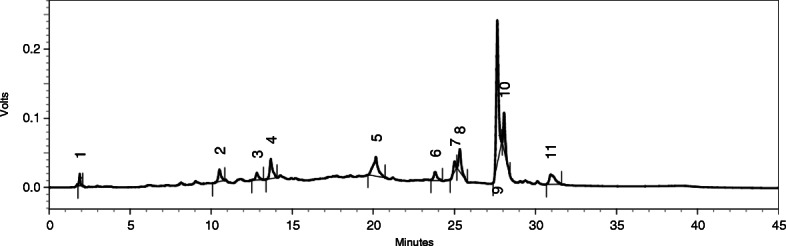
HPLC Chromatogram of POL-6

Qualitative HPLC analysis of POL-6 was performed for further confirmation of our previous experiment by the LC-MS technique. The phytochemical screening was based on observed chromatograms (Fig. 2). Eleven peaks were observed, indicating the presence of 11 major compounds. We assumed that the maximum area should correspond to catechin. The early assumption of three compounds identified from LC-MS might be a single compound or the instrument was unable to detect them due to fewer concentrations of those molecules in the mixture. Different peaks, retention time, peak area, and area percentage was analyzed properly (Table 2). HPLC profiling assured the presence of 11 major compounds in POL-6. For identification, we collected all the markers and performed the HPTLC analysis further.

**Table 2:** Retention time, peak area and area percentage of different peaks observed in HPLC

Sr. No.	Retention Time	Area	Percentage of total area
1.	1.892	90,417	1.90
2.	10.517	201,504	4.23
3.	12.825	136,234	2.86
4.	13.683	343,799	7.22
5.	20.167	593,571	12.46
6.	23.808	171,106	3.59
7.	25.008	118,638	2.49
8.	25.325	352,297	7.39
9.	27.642	1,990,010	41.77
10.	28.058	417,285	8.76
11.	30.942	349,156	7.33

### HPTLC quantification of major constituents present in POL-6

**Fig. 3:**
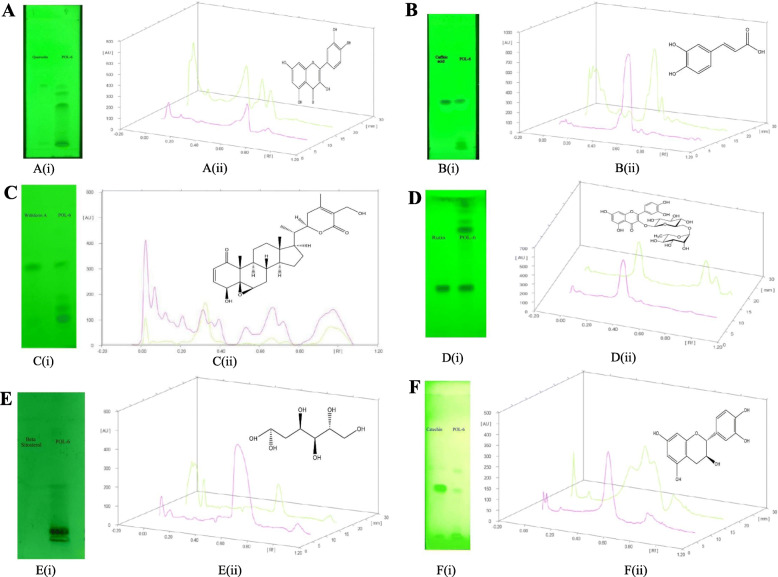
HPTLC chromatogram of POL-6 and all other compounds. **a**. TLC and HPTLC estimation of quercetin in POL-6. a(i). TLC plate of POL-6 with Quercetin. a(ii). HPTLC chromatogram of POL-6 and quercetin. **b.** TLC and HPTLC estimation of Caffeic acid in POL-6. b(i). TLC plate of POL-6 with Caffeic acid. b(ii). HPTLC chromatogram of POL-6 and Caffeic acid. **c**. TLC and HPTLC estimation of Withaferin A in POL-6. c(i). TLC plate of POL-6 with Withaferin A. c(ii). HPTLC chromatogram of POL-6 and Withaferin A. **d.** TLC and HPTLC estimation of Rutin in POL-6. d(i). TLC plate of POL-6 with Rutin d(ii). HPTLC chromatogram of POL-6 and Rutin. **e**. TLC and HPTLC estimation of β-Sitosterol in POL-6. e(i). TLC plate of POL-6 with β-Sitosterol. e(ii). HPTLC chromatogram of POL-6 and β-Sitosterol. **f**. TLC and HPTLC estimation of Catechin in POL-6. f(i). TLC plate of POL-6 with Catechin. f(ii). HPTLC chromatogram of POL-6 and Catechin

HPTLC analysis of POL-6 was performed and a final assurance was done by matching the spots present in the POL-6 with the assumed markers. The R_f_ value of the markers was matched with the compounds in our herbal extract. The HPTLC experimentation revealed the presence of six major compounds in the POL-6 and failed to detect the other five compounds (they might be present in minute quantity). The six identified compounds were quantified using a known protocol. The Mobile phase consisting of Toluene: methanol (7:3, v/v) showed sharp peaks with an Rf value of 0.65 for Quercetin (Fig. 3a). Quercetin found in the POL-6 was 1.50% w/w of POL-6. The Mobile phase consisting of toluene: ethyl acetate: formic acid (5:4:1, v/v/v) revealed sharp peaks with an Rf value of 0.49 for Caffeic acid (Fig. 3b). Caffeic acid found in the POL-6 was 1.059% w/w of POL-6. The Mobile phase consisting of chloroform: methanol (9.5:0.5, v/v) showed sharp peaks with an R_f_ value of 0.59 for Withaferin A (Fig. 3c). Withaferin A found in the POL-6 was 0.921% w/w of POL-6. The Mobile phase consisting of ethyl acetate: formic acid: acetic acid: water (10:1.1:1.1:2.6, v/v/v/v) showed sharp peaks with an Rf value of 0.86 for Rutin (Fig. 3d). Rutin found in the POL-6 was 0.86% w/w of POL-6. The Mobile phase consisting of n-hexane: ethyl acetate (8:2, v/v) showed sharp peaks with an Rf value of 0.61 for β-Sitosterol (Fig. 3e). β-Sitosterol found in the POL-6 was 0.60% w/w of POL-6. The Mobile phase consisting of toluene: ethyl acetate: formic acid (5:4:1, v/v/v) revealed sharp peaks with an Rf value of 0.22 for Catechin (Fig. 3f). Catechin found in the POL-6 was 2.86% w/w of POL-6. HPTLC analysis confirmed that the maximum percentage area in HPLC belonged to Catechin. HPTLC chromatogram of POL-6 and all other compounds are presented in Fig. 3.

### Ethanol consumption and body weight changes of the animals

Ethanol intake by the individual animal in ethanol-fed groups was recorded daily for 15 days and calculated as g/kg/day. Daily ethanol consumption in ethanol-fed groups varied between 13.57 ± 1.85 to 17.12 ± 1.34 g/kg during the exposure to 9% ethanol. No significant difference in ethanol intake was noticed among the ethanol-fed groups. An increase in the bodyweight of approximately 11.2% in control group animals and 6.4% in ethanol-fed animals were observed over the initial body weight at the end of the study.

### Blood alcohol concentration measurement

Blood alcohol concentration (BAC) was measured on day 15th and alcohol withdrawal days 16th, 17th^,^ and 18th and was expressed as mean ± SEM. On the day 15th of the study, BAC was found to be 111.6 ± 8.151 mg %. Very low concentration of alcohol was observed in the animals after 24 h of alcohol withdrawal (8.012 ± 1.325 mg %), 2.017 ± 0.285 mg % after 48 h of alcohol withdrawal, 0.00 ± 0.00 mg % after 72 h of alcohol withdrawal.

### Effect of POL-6 on ethanol withdrawal anxiety-like behavior in the EPM test

**Fig. 4:**
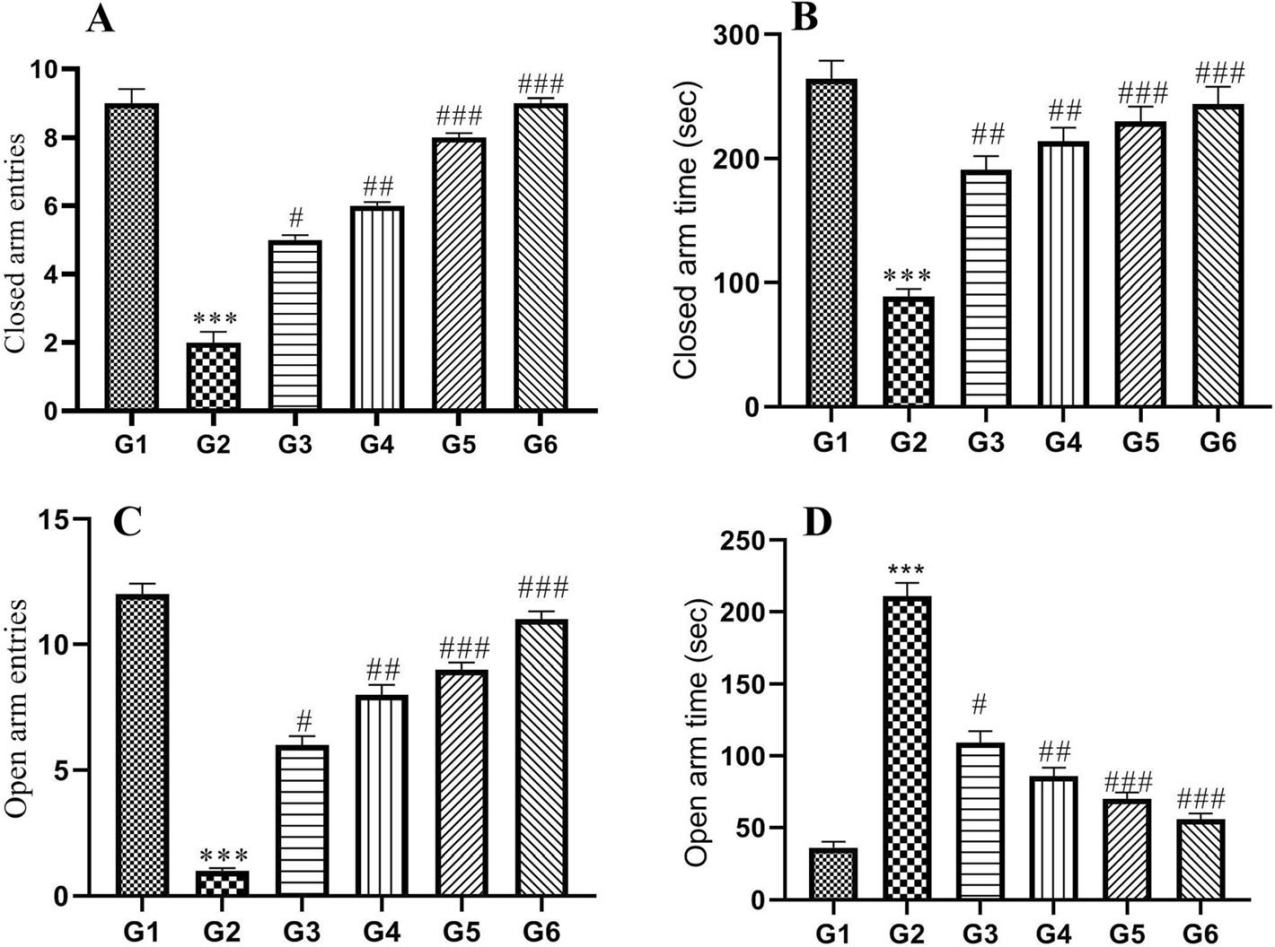
Effect of drug treatment on ethanol withdrawal anxiety when tested on the elevated plus-maze in rats. **a** Number of entries in the closed arm. **b** Time spent in the closed arm. **c** Number of entries in the open arm. **d** Time spent in the open arm. *p < 0.05, **p < 0.01, ***p < 0.001 (Compared to normal control); #*p* < 0.05, ##*p* < 0.01, ###*p* < 0.001 (Compared to disease control); One-way ANOVA; Dunnett’s multiple comparison test. G1-Normal Control, G2-Disease Control, G3-POL-6 treatment (20 mg/kg), G4-POL-6 treatment (50 mg/kg), G5-POL-6 treatment (100 mg/kg), G6-Standard (Diazepam 2 mg/kg)

When tested on the EPM ethanol-fed animals revealed a significant decrease (*p* < 0.001) in the time spend and in the number of entries into the open arms when compared to the normal control group. A significant increase (*p* < 0.001) in the time spent and in the number of entries into the closed arms was also observed in the ethanol-fed animals when compared to the normal group animals (Fig. 4). These findings revealed the development of anxiety in ethanol withdrawal animals. Treatment with POL-6 (20, 50, and 100 mg/kg, oral) and diazepam (2 mg/kg) for three consecutive days produced a significant (*p* < 0.001) increase in the time spend and in the number of entries into the open arms and significant decrease (*p* < 0.001) in the time spend and in the number of entries into the closed arms when compared to the disease control rats (Fig. 4).

### Effect of POL-6 on ethanol withdrawal anxiety-like behavior in light and dark test

**Fig. 5:**
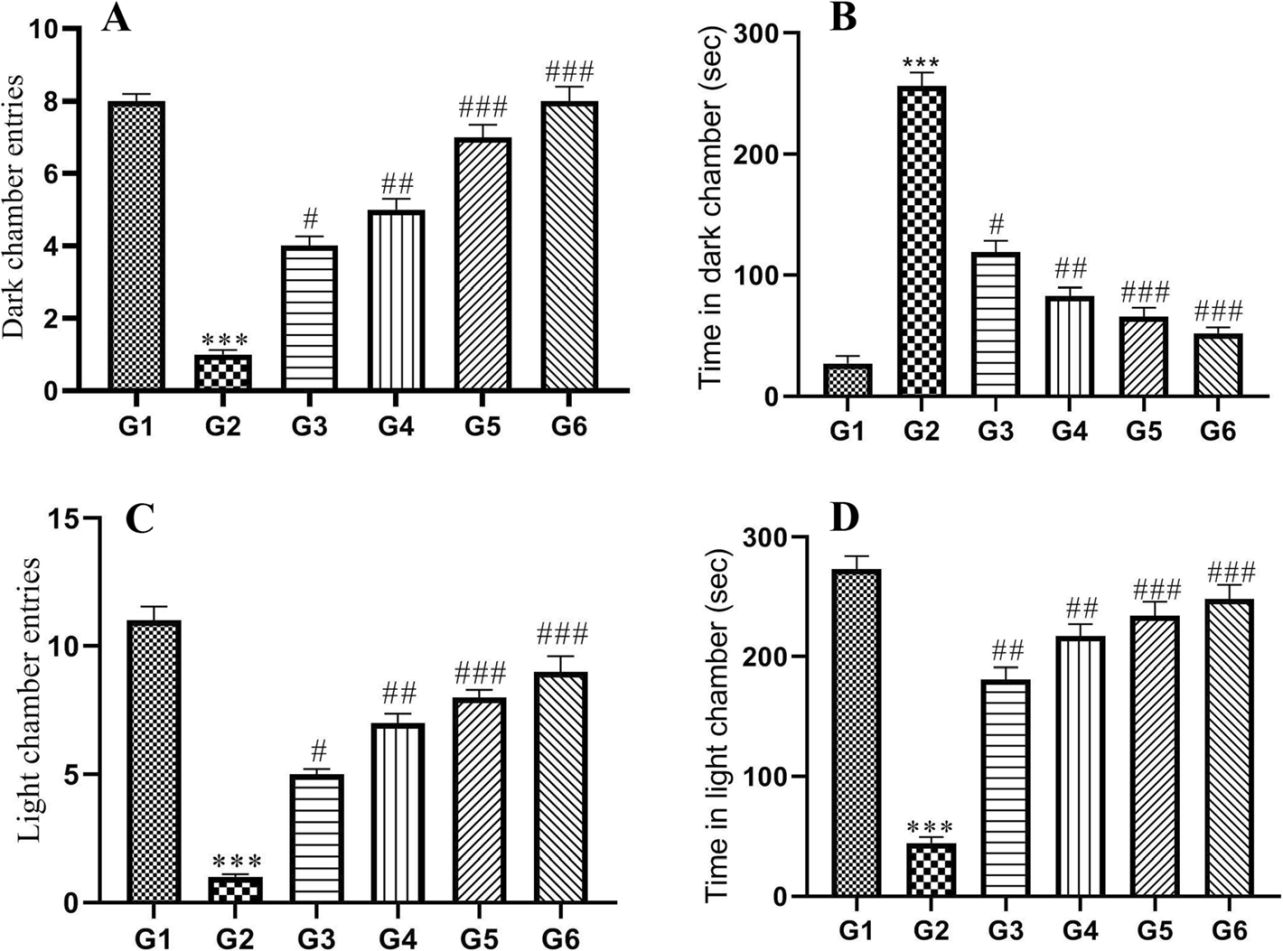
Effect of drug treatment on ethanol withdrawal anxiety when tested on light and dark model in rats. **a** Number of entries in the dark chamber (**b**) Time spent in the dark chamber. **c** Number of entries in the light chamber (**d**) Time spent in the light chamber. *p < 0.05, **p < 0.01, ***p < 0.001 (Compared to normal control); #*p* < 0.05, ##*p* < 0.01, ###*p* < 0.001 (Compared to disease control); One-way ANOVA; Dunnett’s multiple comparison test. G1-Normal Control, G2-Disease Control, G3-POL-6 treatment (20 mg/kg), G4-POL-6 treatment (50 mg/kg), G5-POL-6 treatment (100 mg/kg), G6-Standard (Diazepam 2 mg/kg)

When tested on the light and dark model ethanol-fed animals revealed a significant decrease (*p* < 0.001) in the time spent and in the number of entries into the light chamber of the light and dark model when compared to the normal control animals. A significant increase (*p* < 0.001) in the time spend and in the number of entries into the dark chamber of the light and dark model was also noticed in the ethanol-fed animals when compared to the normal control animals (Fig. 5). Similar to the EPM, the results from the light and dark model revealed the development of anxiety in the ethanol withdrawal animals. Treatment with POL-6 (20, 50, and 100 mg/kg, oral) and diazepam (2 mg/kg) for three consecutive days produced a significant (*p* < 0.001) increase in the time spend and in the number of entries into the light chamber and significant decrease (*p* < 0.001) in the time spend and in the number of entries into the dark chamber when compared to the disease control rats (Fig. 5).

### Effect of POL-6 on biochemical changes induced by alcohol withdrawal in rats

**Fig. 6:**
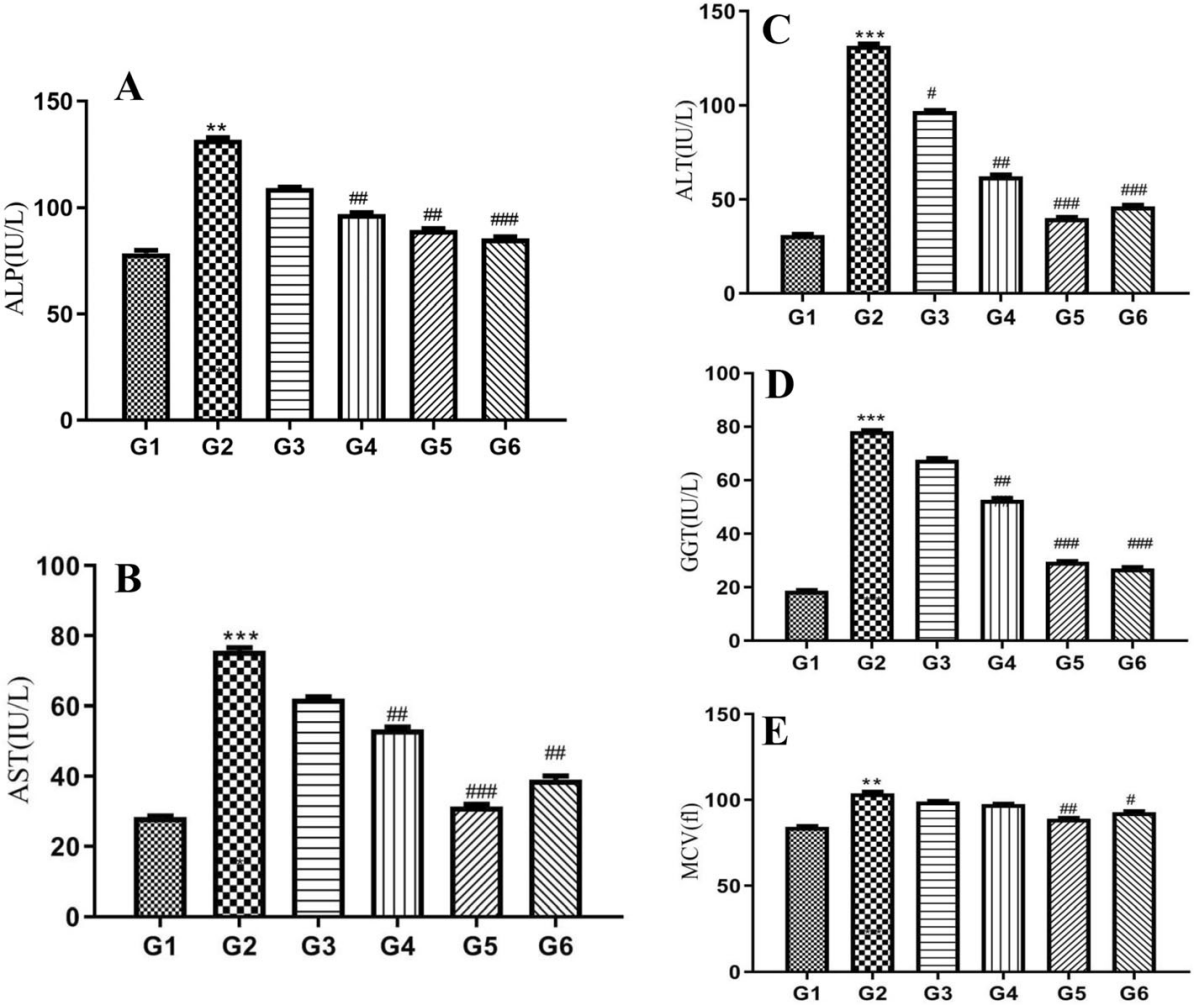
Effects of POL-6 on (**a**) ALP level (**b**) AST level (**c**) ALT level (**d**) GGT level and (**e**) MCV level altered during alcohol withdrawal in rats. *p < 0.05, **p < 0.01, ***p < 0.001 (Compared to normal control); #*p* < 0.05, ##*p* < 0.01, ###*p* < 0.001 (Compared to disease control), Two-way ANOVA, post-hoc Tukey’s multiple comparison test. G1-Normal Control, G2-Disease Control, G3-POL-6 treatment (20 mg/kg), G4-POL-6 treatment (50 mg/kg), G5-POL-6 treatment (100 mg/kg), G6-Standard (Diazepam 2 mg/kg)

The effect of POL-6 on the traditional alcohol markers like ALP, AST, ALT, GGT, and MCV was explored (Fig. 6a, b, c, d, e). Alcohol administration in alcohol-fed animals for the following 15 days significantly (*p* < 0.001) elevated the levels of ALP, ALT, AST, GG T, and MCV when compared to the normal control animals. POL-6 (20, 50, 100 mg/kg, oral) and diazepam (2 mg/kg) treatment for the following 3 days significantly (*p* < 0.001) reversed the elevated levels of ALP, AST, ALT, GGT, and MCV when compared to the disease control animals. The results of alcohol liver markers are shown in (Fig. 6a, b, c, d, e).

### Effect of POL-6 on GABA_A_ receptor subunit gene expression changes in ethanol withdrawal

**Fig. 7:**
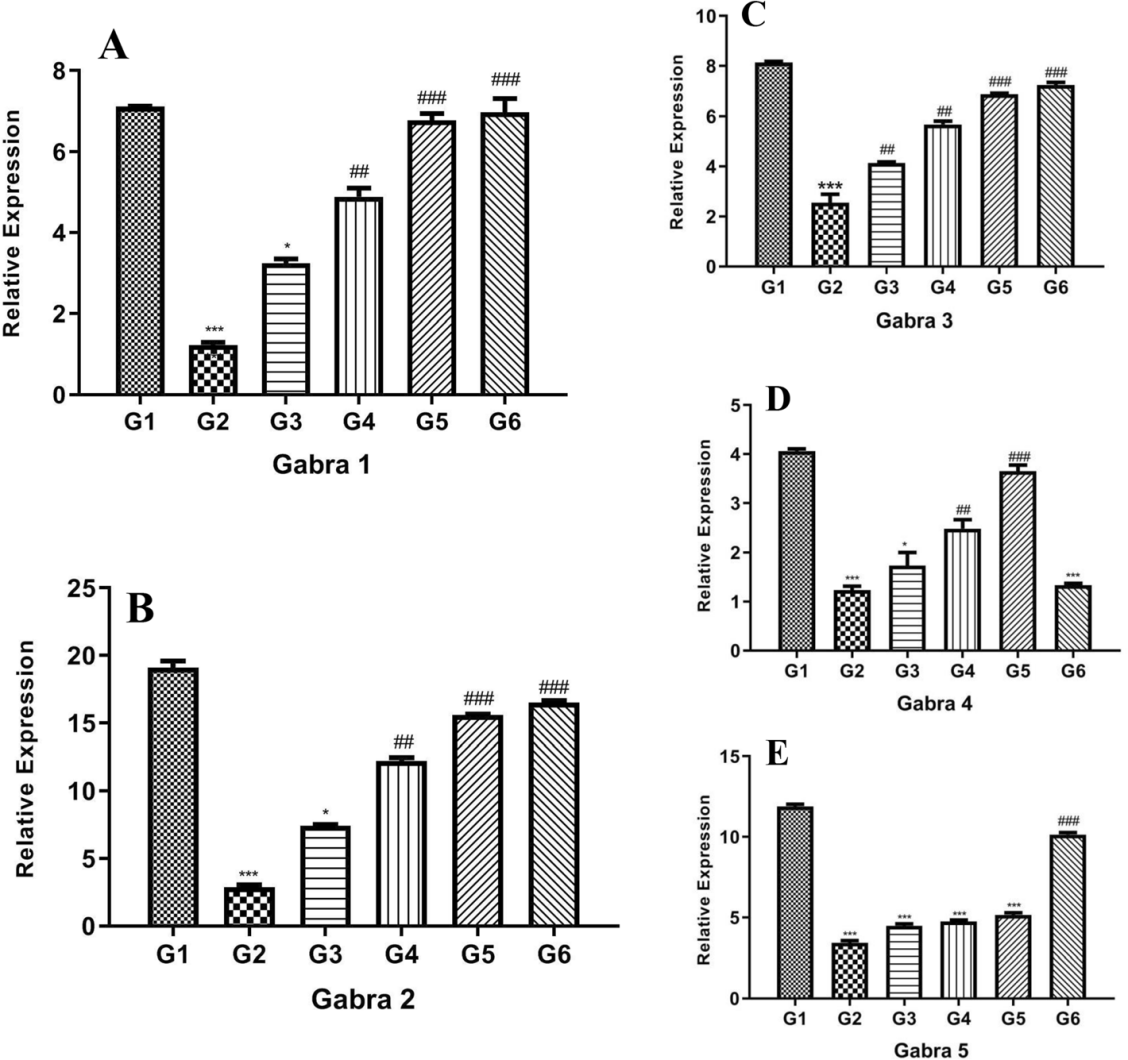
Effect of drug treatment on the relative mRNA expression of GABA_A_ subunits in amygdala of the rats. **a** Gabra 1 (**b**) Gabra 2 (**c**) Gabra 3 (**d**) Gabra 4 (**e**) Gabra 5. *p < 0.05, **p < 0.01, ***p < 0.001 (Compared to normal control); #*p* < 0.05, ##*p* < 0.01, ###*p* < 0.001 (Compared to disease control); One-way ANOVA; Dunnett’s multiple comparison test. G1-Normal Control, G2-Disease Control, G3-POL-6 treatment (20 mg/kg), G4-POL-6 treatment (50 mg/kg), G5-POL-6 treatment (100 mg/kg), G6-Standard (Diazepam 2 mg/kg)

**Fig. 8:**
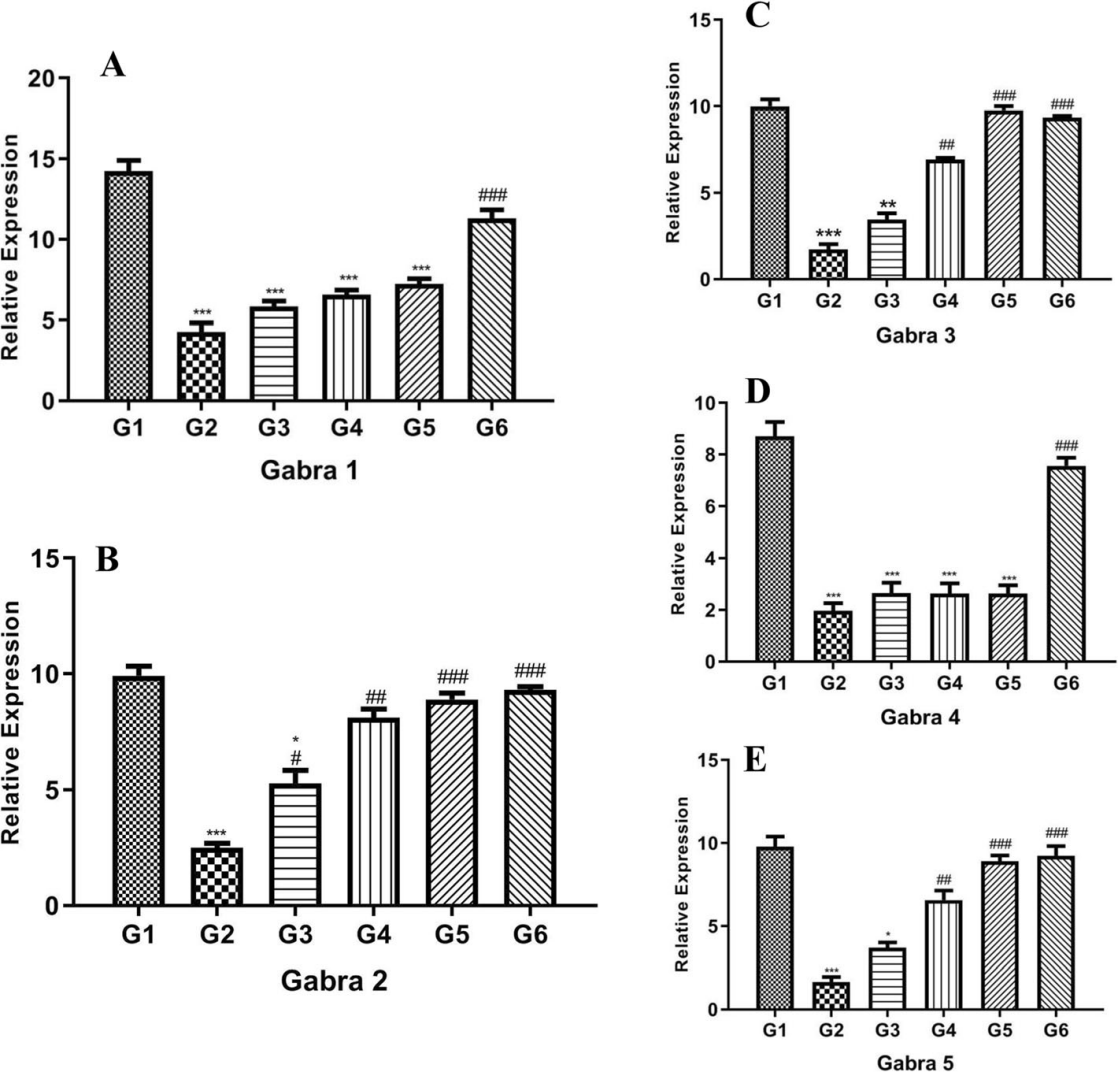
Effect of drug treatment on the relative mRNA expression of GABA_A_ subunits in hippocampus of the rats. **a** Gabra 1 (**b**) Gabra 2 (**c**) Gabra 3 (**d**) Gabra 4 (**e**) Gabra 5. *p < 0.05, **p < 0.01, ***p < 0.001 (Compared to normal control); #*p* < 0.05, ##*p* < 0.01, ###*p* < 0.001 (Compared to disease control); One-way ANOVA; Dunnett’s multiple comparison test. G1-Normal Control, G2-Disease Control, G3-POL-6 treatment (20 mg/kg), G4-POL-6 treatment (50 mg/kg), G5-POL-6 treatment (100 mg/kg), G6-Standard (Diazepam 2 mg/kg)

Figure 7a, b, c, d, and e demonstrates the influences of POL-6 on the altered expression of GABA_A_ receptor subunits in rats’ amygdala. Two-way ANOVA represented the influences of POL-6 on the relative expression of Gabra1, Gabra2, Gabra3, Gabra4, and Gabra 5 in the amygdala. Dunnett’s multiple comparison tests showed that the relative expression of Gabra1, Gabra2, Gabra3, Gabra4, and Gabra 5 were significantly (*p* < 0.001) down-regulated in the disease control rats when compared to the normal control rats. In comparison with the disease group, POL-6 treatment normalized down-regulated genes with concentrations of 50 and 100 mg/kg only in Gabra 1, 2, and 4. However, normalization of Gabra 3 was seen in all three doses (20, 50, and 100 mg/kg). No significant changes were detected in the gene expression of Gabra 5 on the treatment with POL-6 (20, 50, and 100 mg/kg, oral). Diazepam (2 mg/kg) treatment significantly (*p* < 0.001) normalized the down-regulated genes Gabra1, Gabra 2, Gabra3, and Gabra 5 in comparison to the disease control rats, however; no significant changes were detected in the gene expression of Gabra 4 on the treatment with Diazepam (2 mg/kg). Figure 8a, b, c, d, and e demonstrates the influences of POL-6 on the altered expression of GABA_A_ receptor subunits in the rats’ hippocampus. Two-way ANOVA represented the influences of POL-6 on the relative expression of Gabra1, Gabra2, Gabra3, Gabra4, and Gabra 5 in the hippocampus. Dunnett’s multiple comparison tests showed that the relative expression of Gabra1, Gabra2, Gabra3, Gabra4, and Gabra 5 were significantly (*p* < 0.001) down-regulated in the disease control rats when compared to the normal control rats. Normalization of down-regulated genes in comparison to treatment control for all doses of POL-6 (20, 50, and 100 mg/kg, oral) is only seen in Gabra 2 and at 50 and 100 mg in Gabra 2, 3, and 5. No significant changes were detected in the expression of Gabra 1 and Gabra 4 on the treatment with POL-6 (20, 50, and 100 mg/kg, oral). Diazepam (2 mg/kg) treatment significantly (*p* < 0.001) normalized the down-regulated genes Gabra1, Gabra 2, Gabra3, Gabra4, and Gabra 5 in comparison to the disease control rats

## Discussion

Plants are the basis of both traditional medicines and modern drug discoveries. In Ayurveda, the formulation is classified based on two principles: single drug usage or using multiple drugs known as polyherbal preparation or herb-herb combination [32]. In the present study POL-6 preparation containing six plants extracts *Bacopa monnieri, Hypericum perforatum, Centella asiatica, Withania somnifera, Camellia sinesis, and Ocimum sanctum* was studied for its pharmacological beneficial effects in ethanol withdrawal anxiety-like behavior. For the preparation of POL-6 most effective doses of the plants were selected from the literature and POL-6 was prepared by mixing all the plant extracts (*Bacopa monnieri, Hypericum perforatum, Centella asiatica, Withania somnifera, Camellia sinesis, and Ocimum sanctum*) in a ratio of 2:1:2:2:1:2 proportions respectively. Polyherbal preparations containing plant extracts of the different plants are reported to have a diverse group of chemical constituents. Hence in our study phytochemical profiling of POL-6 was initiated with LC-MS analysis. An intense peak was observed at 291.0898 m/z which were found similar to Catechin. Twelve other compounds Withaferin A, Withanolide, Luteolin, β-Setosterol, Quercetin, Hypericin, Rutin, Linalool, Caffeic acid, Eugenol, D-Mannitol, and Withanone were detected in the LC-MS analysis of POL-6. The discrepancy of LC-MS was that we observed a similar mass of Withaferin A, Withanolide, and Withanone. For further authentication, we carried out the HPLC profiling of POL-6. HPLC profiling of POL-6 revealed 11 peaks, indicating the presence of 11 major compounds. HPLC profiling of POL-6 showed that the three compounds (Withaferin A, Withanolide, and Withanone) identified with LC-MS might be a single compound or maybe they were present in fewer concentrations. For further identification, we collected all the markers and performed the HPTLC analysis. The HPTLC study revealed the presence of six major compounds in the POL-6 and failed to detect the other five compounds. Hence phytochemical profiling of POL-6 by LC-MS, HPLC, and HPTLC showed that POL-6 contains six major compounds i.e. Withaferin A, Quercetin, Caffeic acid, β-Sitosterol, Rutin, and Catechin. Quantification through HPTLC also revealed that the maximum percentage area in HPLC belonged to Catechin as Catechin was found to be 2.86% w/w of POL-6.

The most appropriate model for exploring ethanol withdrawal syndrome in animals is the ethanol administration in a liquid diet or a two-bottle choice drinking paradigm model. The latter model is proven to be clinically relevant to mimic the human condition as the animals can voluntarily consume either ethanol or water [33, 34]. Previous studies reported dependence and abstinence to alcohol occurs in rats at daily consumption of ethanol over 9 g/kg for 15 days continuously [35, 36]. The two-bottle choice drinking paradigm model was selected in our study. An increase in the bodyweight of the rats approximately 11.2% in control group animals and 6.4% in ethanol-fed animals were observed over the initial body weight at the end of the study. The body weight in ethanol-fed rats changes lightly during the study as compared to the normal control rats. Earlier studies reported that alcohol decreases the secretion of digestive enzymes and affects absorption, metabolism, and excretion of essential nutrients [37]. Hence alcohol consumption by the alcohol-fed animals could be the possible reason for a slight change in body weight in comparison to control animals. Ethanol intake by the individual rat in ethanol-fed groups was also recorded daily during the study and calculated as g/kg/day. Daily ethanol consumption in ethanol-fed groups varied between 13.57 ± 1.85 to 17.12 ± 1.34 g/kg during the exposure to 9% ethanol. To see whether the alcohol liquid diet, will achieve reliable BACs and also confirm clearance of alcohol during withdrawal periods BACs assay was performed. BACs results in our study indicated the complete clearance of ethanol concentration after 72 h of alcohol withdrawal. Previous studies reported that BACs in the range of 80 to 132 mg % during alcohol intake results in ethanol dependence [38]. In our study, BAC was found to be 111.6 ± 8.151 mg % on the 15th day which showed ethanol dependence in animals. We further investigated the effect of POL − 6 on ethanol withdrawal anxiety in rats. Anxiety is the most important negative motivator to experience the same level of the rewarding effects of alcohol [39]. Alcohol withdrawal anxiety results in adaptive adjustments in the brain areas such as the amygdala and hippocampus associated with changes in many neuropeptides, neurotransmitters, and hormonal systems. In the present study, a high level of anxiety was observed on the 3rd day of ethanol withdrawal. Hence, we explored the effect of POL-6, diazepam, and vehicle, in the EPM and LDT on the 18th day only. EPM and LDT are well-known tests used for exploring the antianxiety effects of the drugs. EPM is the most widely used apparatus used to assess exploration, anxiolytic responses, and motor behavior. Rodents display approach-avoidance conflict which is stronger in exposed open areas and preference to enclosed arm, therefore, spending more time in the enclosed arm. When the animal enters the open arm they freeze and show fear [40]. Drugs that relieve anxiety increase the time spent and the number of entries in open arms. In the present study, disease (ethanol withdrawal) control group animals spent less time in the open arms exploration, and the time spent in closed arms was more as compared to normal control animals. POL-6 (20, 50, and 100 mg/kg, oral) and diazepam (2 mg/kg) treatment given for the following 3 days increased the number of entries and the time spent by the alcohol-fed rats in the open arm. This shows the anxiolytic effects of the POL-6 in the rats. LDT test is a useful model to study anxiolytic activity. The brightly lit compartment represents a destructive environmental stressor that reduces the normal exploratory behavior of rats. Exploratory behavior from one compartment to another and the time spent in each compartment are parameters reported in anxiety. In the present study, there was an inhibition of anxiety behavior, latency to the dark chamber by the animals was decreased, and numbers of transitions in both the compartments and time spent in the light chamber were also decreased showing the anxiety amid alcohol withdrawal. Treatment with POL-6 (20, 50, and 100 mg/kg, oral) and diazepam (2 mg/kg) for the following 3 days increases the time spent and the number of entries in the light compartment by the rats. During ethanol withdrawal anxiety, there is a decline in the inhibition of excitatory activity by GABA_A_ receptors that leads to CNS hyperexcitability [41]. Therefore, stimulation at a normal level even can cause over-excitation due to the reduced suppression of the CNS, thus alcohol withdrawal-induced anxiety was observed. The individual constituents present in the POL-6 have been reported previously for their antianxiety activity at high doses, and then the need for the development of polyherbal preparation lies in Ayurveda which states that when the drugs are combined they show the potentiation of response even at low doses [42]. POL-6 might have potentiated the anxiolytic action as *Withania somnifera* is proven to have GABA mimetic activity, moreover; *Withania somnifera* has evidence to reduce the levels of mediators that cause anxiety [43]. *Camellia sinensis* has a stimulating effect on the brain due to its methylxanthine content that antagonizes adenosine thus ameliorating the ethanol withdrawal state [44]. *Ocimum sanctum* also reported decreasing the levels of anxiogenic mediators in the brain also called anti stressor and reported to possess antioxidant activity [45]. Thus we can conclude that herb-herb combination in the POL-6 proven to be beneficial in ameliorating alcohol withdrawal anxiety in rats. ALP, ALT, AST, GGT, and MCV are utilized in clinical practice as traditional alcohol markers to identify chronic heavy drinking [46]. Excessive alcohol consumption is reported to increase the GGT level in the serum that is one of the causes of oxidative stress [47]. In our studies, we have quantified the six major compounds through HPTLC in POL-6. These all compounds Quercetin, Withaferin A, β-sitosterol, Catechin, Rutin, and Caffeic acid are reported to reduce oxidative stress. Hence, the potential antioxidant property of POL-6 might have brought about the inversion of all the mentioned alcohol biomarkers.

Further, we explored the influences of POL-6 on the mRNA expression of GABA_A_ receptor subunits. GABA_A_ receptor allosteric binding sites are targets for alcohol that modulate GABAergic function. Alcohol targets GABA_A_ receptors because they are key inhibitory neurotransmitters in the CNS and play a central role in mediating the consequences of ethanol. GABA_A_ receptors have many subunit isoforms generally α1- α5 subunits. The gene which encodes the Gabra subunits is reported to have remarkable plasticity in alcohol addiction. Reduction in GABAergic transmission and GABA_A_ receptors down-regulation has been reported in alcohol withdrawal [48]. GABA_A_ receptors down-regulation in alcohol withdrawal develops a hyperglutamatergic state, which in combination with reduced GABA function leads to excessive excitatory signaling, resulting in alcohol withdrawal anxiety [49]. In our study, we found that alcohol withdrawal after 15 days of alcohol utilization in rats emanated a decrease of Gabra1, Gabra2, Gabra3, Gabra4, and Gabra5 gene expression in the hippocampus and amygdala of the rats. Interestingly, treatment with POL-6 (50 and 100 mg/kg, oral) for the following 3 days normalized the down-regulated Gabra1, Gabra 2, Gabra 3 and Gabra 4 gene expression in the amygdala; Gabra2, Gabra3 and Gabra5 gene expression in the hippocampus of the rats however normalized effect with low dose treatment of POL-6 (20 mg/kg) was observed only in the gene expression of Gabra 3 in the amygdala and with Gabra 2 in the hippocampus. Similarly, no effect was observed on the down-regulated gene expression of Gabra5 in the amygdala and Gabra1 and Gabra4 in the hippocampus of the rats with any of the POL-6 treatment. Another group treatment with Diazepam (2 mg/kg) for the following 3 days normalized the down-regulated Gabra1, Gabra2, Gabra3, and Gabra5 gene expression in the amygdala, Gabra1, Gabra 2, Gabra3, Gabra4 and Gabra5 gene expression in the hippocampus of the rats. However, no effect was seen on the down-regulated gene expression of Gabra4 in the amygdala of the rats with diazepam treatment. *Withania somnifera*, *Hypericum perforatum*, *Oscimum sanctum*, *Camellia sinesis* are reported to have GABA mimetic effects [50–52]. The constituents present in POL-6 are also reported to modulate GABAergic functions. Quercetin is reported to regulate GABAergic transmission [53]. Rutin is reported to modulate GABA_A_ receptors and increase GABAergic neurotransmission in the amygdala [54]. Withaferin A is reported to have GABAergic activity [55]. Catechin is reported to modulate GABAergic neurotransmission [56]. GABA mimetic effects of the plant extracts and constituents present in POL-6 may have caused normalization of the genes of GABA_A_ receptor subunits in ethanol withdrawal. Hence our findings conclude that GABA mimetic effects of POL-6 normalizes Gabra1, Gabra2, Gabra3, Gabra4, and Gabra5 genes of GABA_A_ receptor subunits in rats amygdala and hippocampus and showed protective effects during ethanol withdrawal anxiety especially at higher concentrations 50 and 100 mg/kg/b.wt.

## Conclusion

The results from the present finding showed that POL-6 possesses a protective effect on alcohol withdrawal anxiety in rats. Gene expression studies on the isolated brain tissues showed that POL-6 normalizes the GABAergic transmission in the amygdala and hippocampus of the rats and inhibits the ethanol withdrawal anxiety-like behaviors. Therefore we concluded that POL-6 may have therapeutic potential for treating ethanol-type dependence as it suppresses ethanol withdrawal anxiety-like behavior.

## Data Availability

All the data obtained and materials analyzed in this research are available with the corresponding author.

## Abbreviations

GABA: γ-aminobutyric acid
NOAEL: No observed adverse effect levels
LC-MS: Liquid chromatography-mass spectroscopy
HPLC: High Performance Liquid Chromatography
HPTLC: High performance thin layer chromatography
TLC: Thin-layer chromatography
CMC: Carboxymethyl cellulose
BAC: Blood alcohol concentration
ALT: Alanine transaminase
AST: Aspartate transaminase
ALP: *Alkaline phosphatase*
GGT: Gamma-glutamyltransferase
EDTA: Ethylene-diamine-tetraacetate
MCV: Mean corpuscular volume
RT-PCR: Real-time quantitative polymerase chain reaction
EPM: Elevated plus maze
LDT: Light and dark test
